# Clinical outcomes of bulky parotid gland cancers: need for self-examination and screening program for early diagnosis of parotid tumors

**DOI:** 10.1186/s12885-021-07902-9

**Published:** 2021-02-18

**Authors:** Sung Yong Choi, Eunkyu Lee, Eunhye Kim, Man Ki Chung, Young-Ik Son, Chung-Hwan Baek, Han-Sin Jeong

**Affiliations:** Department of Otorhinolaryngology - Head and Neck Surgery, Samsung Medical Center, Sungkyunkwan University School of Medicine, Seoul, Republic of Korea

**Keywords:** Parotid gland, Tumor, Surgery, Advanced stage, prognosis

## Abstract

**Background:**

Early detection and diagnosis of parotid gland cancer (PGC) are essential to improve clinical outcomes, because Tumor-Node-Metastasis stage at diagnosis is a very strong indicator of prognosis in PGC. Nevertheless, some patients still present with large parotid mass, maybe due to the unawareness or ignorance of their disease. In this study, we aimed to present the clinical outcomes of bulky PGC (defined by a 4 cm cutoff point for T3–4 versus T1–2 tumors), to emphasize the necessity of a self-examination tool for parotid gland tumor.

**Methods:**

We retrospectively reviewed 60 consecutive cases with bulky (equal to and greater than 4 cm in the longest diameter, determined radiologically) malignant tumors arising from the parotid gland from 1995 to 2016. The clinical and pathological factors were analyzed to identify risk factors for poor outcomes using Cox proportional hazard models. In addition, we designed a self-examination tool for parotid gland tumors, similar to breast self-examination for breast cancer detection.

**Results:**

Patients with bulky parotid cancer showed 48.9% 5-year and 24.5% 10-year overall survival rates and a 47.9% risk of high-grade malignancy. The common pathological diagnoses were carcinoma ex pleomorphic adenoma (18.3%), adenocarcinoma (16.7%), mucoepidermoid carcinoma (16.7%), salivary duct carcinoma (16.7%), and adenoid cystic carcinoma (11.7%). Survival analyses revealed that tumor size (hazard ratio, HR = 1.262 upon increase of 1 cm, 95% confidence interval, 95%CI 1.059–1.502), lymph node metastasis (HR = 2.999, 95%CI 1.048–8.583), and high tumor grade (HR = 4.148, 95%CI 1.215–14.154) were independent prognostic factors in multivariable analysis. Functional preservation of the facial nerve was possible only in less than half of patients.

**Conclusion:**

In bulky PGC, lymph node metastasis at diagnosis and high tumor grade indicated poor survival outcomes, and functional outcomes of the facial nerve were suboptimal. Thus, a public effort seems to be necessary to decrease these patients with bulky PGC, and to increase patients’ self-awareness of their disease. As a way of early detection, we proposed a parotid self-examination tool to detect parotid gland tumors at an early stage, which is similar to breast self-examination.

**Supplementary Information:**

The online version contains supplementary material available at 10.1186/s12885-021-07902-9.

## Background

Malignant tumors arising from the parotid gland are uncommonly seen and represent less than 3% of head and neck cancers [1]. They are more than 20 different histopathologic subtypes of this cancer and histologic grade is universally recognized as an important prognostic factor [2]. However, patients with early-stage parotid gland cancers (PGC) are expected to have relatively favorable prognosis with the current standard treatment modalities, even in those with high-grade tumors [3]. Thus, tumor-node-metastasis (TNM) stage at diagnosis can be the most essential factor for outcome and prognosis in these patients [4]. This finding suggests that early detection and diagnosis of PGC are critical for improving patient survival and treatment outcome.

Most patients with parotid gland tumors present with a painless palpable mass in their parotid gland, although they are often asymptomatic and lesions may be found incidentally. Recently, increasing incidence of major salivary gland cancer measuring 0 to 2.0 cm in diameter was noticed, although there was no significant change in incidence of tumors larger than 2.0 cm in diameter [5]. One of the potential reasons for this epidemiological trend is that early detection and management of parotid gland tumors may increase the incidence of small PGC. However, some patients dismiss the clinical importance of large tumor formation around the parotid gland, and miss an opportunity for cure with fewer complications.

Previously, many studies have indicated that tumor size, grade and metastasis at diagnosis are significant determinants for prognosis, in salivary gland cancer and PGC [6–12]. However, there is little of clinical data about risk of high-grade malignancy, treatment outcomes and treatment-related complications, particularly in advanced T status PGC. Along with the survival rates of bulky PGC patients as the primary endpoint of this study, we also investigated the secondary outcomes, that is, the risk of high-grade malignancy and the functional outcomes (facial nerve function). In addition, we aimed to suggest a screening program (parotid self-examination tool) to detect PGC at an early stage.

## Methods

### Study subjects

This study was a retrospective analysis of consecutive cases, which met the inclusion criteria. The study protocol was approved by our institutional review board (2018–08-083) and the requirement for obtaining informed consent was waived because of the retrospective nature of the study. The clinical data used in the study were de-identified.

In the Tumor-Node-Metastasis (TNM) staging system (American Joint Committee on Cancer staging manual, 7th edition), a cutoff of 4 cm in the long axis is a point to differentiate T1–2 tumors from T3–4 tumors. Thus, we set a 4 cm in diameter as a cutoff point in this study, to evaluate the clinical outcomes of the large tumors (equal to or more than 4 cm in diameter) [13]. Inclusion criteria of the present study were patients older than 18 years, bulky (equal to and greater than 4 cm in the longest diameter, determined radiologically) malignant tumor arising from the parotid gland, and proper integrity of medical records. Exclusion criteria were patients with previous head and neck cancers and with metastasis to parotid glands.

**Fig. 1:**
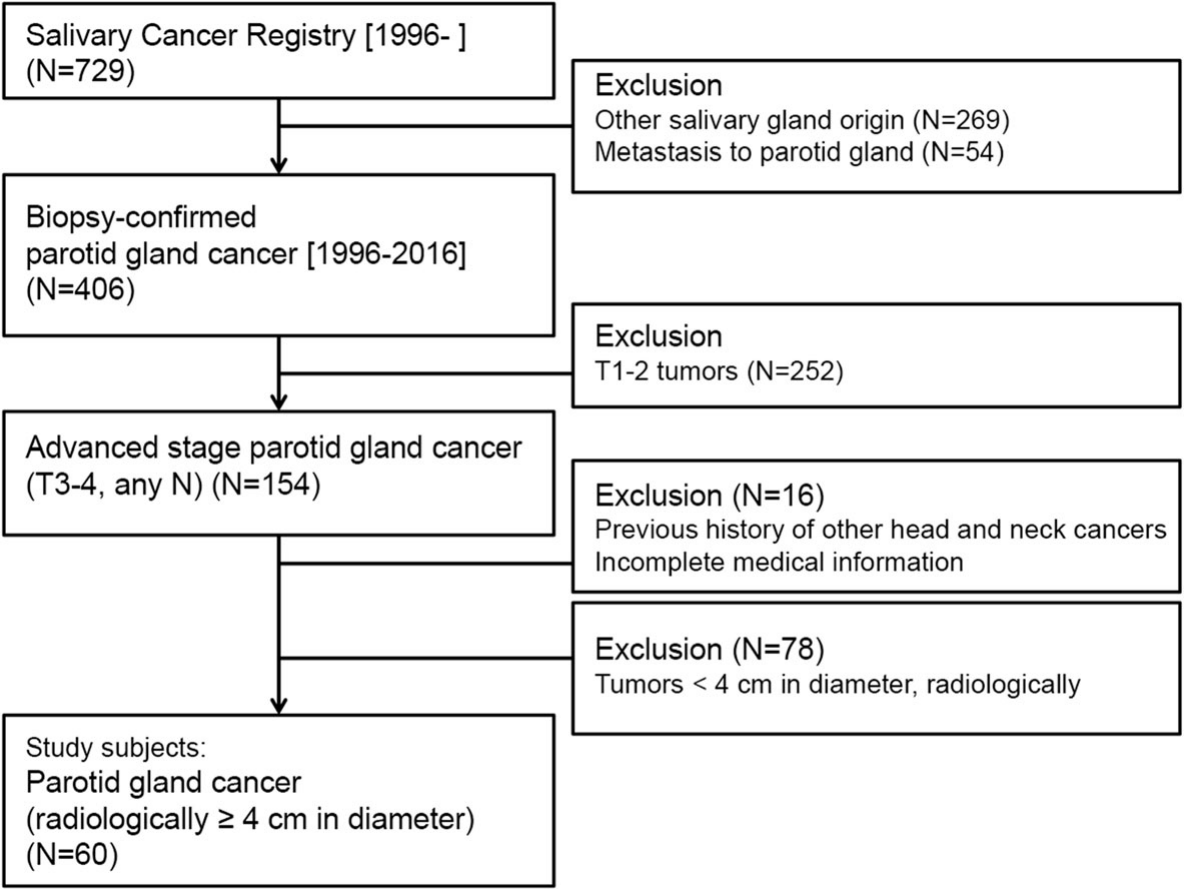
A flow chart of subject enrollment

From the salivary gland cancer registry of our institute between January 1995 and December 2016, we identified 406 biopsy-confirmed PGC subjects. Then, we collected only T3–4 tumors, and excluded the patients with previous history of other head and neck cancers and with tumors less than 4 cm in diameter. Finally, a total of 60 patients met the inclusion criteria, and their data were analyzed retrospectively (Fig. 1).

### Preoperative work-up and pathological diagnosis

All subjects preoperatively underwent head and neck contrast-enhanced computed tomography (CT) and pre-operative cytology or core needle biopsy (ultrasonography-guided if indicated). CT scans were performed with non-contrast axial view (14.5 mm thickness), contrast-enhanced axial view (8 mm thickness) and contrast-enhanced coronal view (3.6 mm thickness). Magnetic resonance imaging (MRI) and positron emission tomography for tumor characterization and metastasis evaluation were also conducted, if high-grade tumor was suspected. All CT or MRI images were interpreted by two board-certified radiologists having five and ten years of experience in reading head and neck images, respectively. Both reviewers were blinded to the clinical information. If there was a discrepancy in interpretation or measurement, a joint decision was made. Tumor size was presented as the longest diameter of the tumor (cm), and determined radiologically. Considering the potential variation (or error) in measurement, we included a tumor equal to 4 cm in diameter in this study. There was no case of multiple tumors.

A senior pathologist with over 10 years of experience in pathological diagnosis of salivary gland tumors reviewed the pathology slides and reports. Based on surgical pathology reports, each tumor was assigned to a pathological tumor-node- metastasis (pTNM) stage using the 7th American Joint Committee on Cancer staging manual [13]. Clinical staging (cT/cN) was applied in patients who had received non-surgical treatment.

### Risk of high-grade malignancy

Risk of high-grade malignancy was defined as a frequency rate by dividing the number of high-grade cancer cases by the total number of malignant cases in each category (tumor size in the greatest diameter and T classification). For this analysis, we included all parotid gland malignant tumors diagnosed during the same study period [1995–2016], not limited to tumors of more than 4 cm in diameter (*N* = 406).

### Treatment modalities

The extent of surgery was divided into total, superficial, or partial parotidectomy. Neck dissection was performed for clinically suspicious lymph node metastasis (therapeutic neck dissection) or high-grade tumors suggested on preoperative work ups (elective neck dissection). Therapeutic neck dissection included ipsilateral neck lymph node levels 1 to 5 and elective neck dissection was targeted for levels 1b to 3, and infra-parotid lymph nodes. During the study period, four surgeons (having more than 5 years of head and neck surgery experience, and treating more than 15 to 20 cases of parotidectomy every year) had conducted the parotidectomies. No difference was found in terms of postoperative complications including facial weakness among responsible surgeons.

As an adjuvant treatment, post-operative radiation therapy (RT) was applied to all surgical patients (60–66 Gy by 2.0–2.2 Gy per fraction over 5.5–6 weeks). In adjuvant RT, clinical target volume included the primary tumor bed and pathologically involved regional lymphatics with adequate margins. Elective neck irradiation was determined on an individual basis considering the estimated risk of metastasis based on location, histologic type, extent, and grade of primary tumor. RT was delivered with 4- or 6-MV photons generated from a linear accelerator.

For definitive RT, gross tumor volume was defined as volume of primary tumor and involved lymph nodes based on all available clinical information. The clinical target volume of primary tumor was delineated by adding 5 mm margins in all directions from gross tumor volume, and the margins were optionally modified in accordance with the anatomic boundaries of the tumor location and/or the adjacent organs. Chemotherapy was administered concurrently with radiation or independently in the palliative setting. Cisplatin was the major drug for chemotherapy, in combination with other drugs depending on medical oncologist decision and clinical situation. In this study, we had two radiation oncologists and two medical oncologists, who were specialized for the management of head and neck cancer and had more than 5 years of experience in their fields.

### Oncological and functional outcomes

The primary endpoints were overall survival (OS) and disease-free survival (DFS) in all patients and in patients with resectable parotid gland cancers. OS and DFS were calculated as the time elapsed from the initiation of treatment or the date of diagnosis until the time of any death and recurrence, respectively. Patients without any events (death or recurrence) at the last clinical follow-up were censored. The final oncological status was divided into no evidence of disease, alive with disease (loco-regional or distant disease), cancer-related or unrelated death.

Facial nerve palsy at diagnosis was evaluated clinically based on three subsites: forehead, eye, and lip. For statistical purpose, we counted a patient who had suffered from facial weakness in one or more subsite as a case of facial nerve palsy, regardless of severity. Preservation of facial nerve was confirmed if there was no tumor invasion and no sacrifice of any branches of nerve during surgery.

### Statistical analyses

Baseline variables at diagnosis of parotid gland cancers (age, gender, facial nerve palsy, tumor size, tumor grade, presence of nodal metastasis, distant metastasis at diagnosis) were considered as the variables for predicting the outcome. In addition, treatment variable (surgery or not) was also included as a variable. Categorical variables were stratified into two groups. Age and tumor size were calculated as continuous variables. Survival curves were estimated using the Kaplan–Meier method, and group differences were tested using the log-rank test. Prognostic significance of variables was assessed by univariable and multivariable analyses using the Cox proportional hazard model. Statistical analyses were performed using SPSS version 20.0 (IBM Corporation, Armonk, NY, USA). All tests were two-sided and *P* < 0.05 indicated statistical significance.

## Results

### Risk of high-grade malignancy

First, we investigated the risk of high-grade malignancy using a cohort of malignant parotid gland tumors (*N* = 406) to estimate the pathological significance of bulky parotid cancers. The result showed that risk of high-grade malignancy was significantly increased in malignant tumors over 4 cm in dimension (approximately 50.0% of bulky malignant tumors were high-grade tumor) (Table 1). Similarly, advanced T status in malignancy had increased risk of advancing to high-grade malignancy.

**Table 1:** Risks of high-grade malignancy according to tumor size and T classification in malignant parotid gland tumors

Tumor size (greatest dimension)	Total (No.) (*N* = 406)	High-grade malignancy (No.,%) (*N* = 128)	*P*
< 2 cm	109	22 (20.2)	0.0004
2–4 cm	189	65 (34.4)	
≥ 4 cm	73	35 (47.9)	
Unknown	33	6	
T classification			
T1	96	15 (15.6)	< 0.0001
T2	132	41 (31.1)	
T3	61	17 (27.9)	
T4	93	55 (59.1)	
Unknown	24		

### Clinical features of bulky parotid gland cancers

Next, we focused on 60 patients who had bulky (equal to and greater than 4 cm in the longest diameter) malignant tumors arising from the parotid (Table 2). There were 40 males (66.7%) and 20 females (33.3%) with a median age of 58.0 years (interquartile range: 47–71 years). The median tumor size was 4.3 cm (interquartile range: 4–6 cm). In all patients, 16 (26.7%) had facial nerve weakness at diagnosis. Primary tumor extent was classified as T3 in 20 patients (33.3%) and T4 in 40 (66.7%).Half of patients showed regional lymph node metastasis (53.3%), and 8 patients (13.3%) had distant metastasis.

**Table 2:** Baseline characteristics of the patients with bulky parotid cancer (equal to or greater than 4 cm in the longest diameter)

Characteristics (*N* = 60)	No.(%)
Age (median, with interquartile range, years)	58.0 [47.0–71.0]
Sex (Male:Female)	40:20 (66.7:33.3)
Tumor size (longest diameter)^a^ (median with interquartile range, cm)	4.3 [4.0–6.0]
Facial nerve palsy at diagnosis	16 (26.7)
TNM stage^b^	
T3:T4	20:40 (33.3:66.7)
N0:N1:N2:N3	28:7:24:1 (46.6:11.6:40.0:1.7)
M0:M1	52:8 (86.7:13.3)
Tumor pathology	
Carcinoma ex pleomorphic adenoma	11 (18.3)
Adenocarcinoma not otherwise specified	10 (16.7)
Mucoepidermoid carcinoma	10 (16.7)
Salivary duct carcinoma	10 (16.7)
Adenoid cystic carcinoma	7 (11.7)
Acinic cell carcinoma	3 (5.0)
Myoepithelial carcinoma	2 (3.3)
Others^c^	7 (11.7)
Tumor grade	
High grade	26 (43.3)
Intermediate grade	2 (3.3)
Low grade	29 (48.3)
Unknown	3 (5.0)
Treatments	
Surgery with adjuvant RT or CCRT	47 (78.3)
RT or CCRT (with salvage surgery)	11 (18.3)
Chemotherapy or no treatments	2 (3.3)
Survival outcomes	
No evidence of disease	31 (51.7)
Alive with disease	6 (10.0)
Cancer-related death	22 (36.6)
Unrelated death	1 (1.7)
Follow-up period (median, range, months)	18.2 [0.7–200.2]

^a^ Tumor size determined by radiology findings

^b^ *AJCC* American Joint Committee on Cancer staging system 7th edition

^c^ Carcinoma subtype not determined due to lack of surgical pathology

*RT* Radiation treatment, *CCRT* Concurrent chemo-radiation

In all patients, pathological diagnosis was based on surgical pathology or biopsy. Carcinoma ex pleomorphic adenoma (18.3%) was the most common histologic subtype, followed by adenocarcinoma (16.7%), mucoepidermoid carcinoma (16.7%), and salivary duct carcinoma (16.7%). There were 26 high-grade tumors in this cohort (43.3%). Forty-seven patients (78.3%) underwent surgical resection with adjuvant RT, and 11 patients (18.3%) were initially treated with RT or concurrent chemo-RT. During the follow-up period (median 18.2 months, range 0.7–200.2 months), there were 22 disease-specific deaths (36.6%) and 1 unrelated death (1.7%).

Next, we analyzed the clinical data of 49 patients who underwent initial surgery (*N* = 47) or salvage surgery for curative intent (*N* = 2) (Table 3). Total parotidectomy was performed in 39 patients (79.6%). Neck lymph node dissection was performed in 35 patients (71.6%), of which 15 had pathologically positive lymph nodes. Facial nerve was sacrificed in 31 patients (63.3%): 22 cases in the main trunk, and 9 cases in peripheral branches. Among them, there were 9 cases that underwent nerve grafting (sural nerve) (15.0%). Decision for soft tissue reconstruction was made by surgeons, based on defect types, patient comorbidity, previous treatment history and availability of reconstructive flap. Regional flaps were used in 11 patients (22.5%), free flap in 5 patients (10.1%), and local flaps in 2 patients (4.1%).

**Table 3:** Surgical characteristics of bulky parotid gland cancers with surgery (*N* = 49)

Characteristics (*N* = 49)	No.(%)
Extent of surgery for primary tumors	
Total parotidectomy	39 (79.6)
Superficial or partial parotidectomy	10 (20.4)
Neck dissection	
No	14 (28.6)
Yes	35 (71.6)
pN(+):pN0	15:20
Perineural invasion (pathological)	
No	38 (77.6)
Yes	11 (22.4)
Preservation of the facial nerve	
Preserved	18 (36.7)
Sacrifice	31 (63.3)
Total (main trunk):partial (branches)	22:9
Facial nerve graft	9 (15.0)
Reconstruction	
No	31 (63.3)
Local flap	2 (4.1)
Regional flap	11 (22.5)
Free flap	5 (10.1)

### Oncological outcomes and survival analysis

**Fig. 2:**
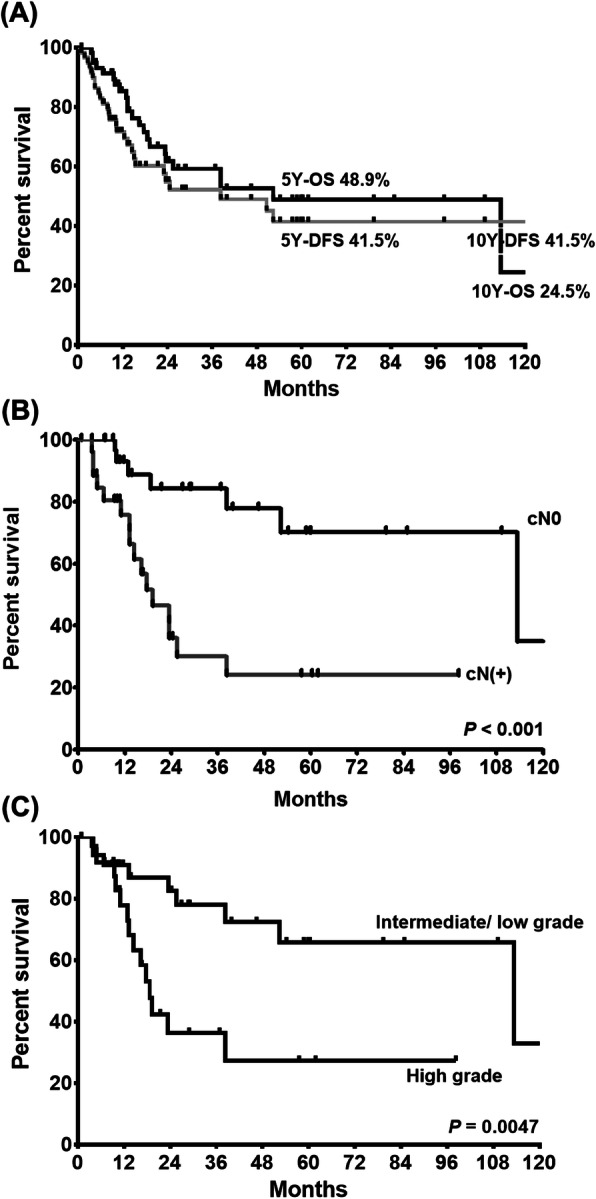
Survival plots of patients with bulky parotid gland cancer. **a** Overall survival (OS) (*N* = 60) and disease-free survivals (DFS) (*N* = 49 with curative surgery). **b** Survival plots stratified by cN and (**c**) tumor grade of enrolled subjects

Five-year OS (*N* = 60) and DFS (*N* = 49) were 48.9 and 41.5%, and those for 10 years were 24.5 and 41.5%, respectively (Fig. 2a). To identify the major prognostic factors in these patients, we conducted univariable and multivariable analyses using clinical and pathological variables (Table 4). Univariable analysis revealed that age, tumor size, lymph node metastasis, tumor grade, and surgery were significant prognostic factors for OS. Kaplan-Meier survival analyses also showed clear discrimination of the survival plots according to nodal status and tumor grade (Fig. 2b-c).

**Table 4:** Cox proportional hazard models for predicting overall survival (*N* = 60)

Variables	Category	Univariable analysis				Multivariable analysis #1^c^				Multivariable analysis #2^c^			
		HR	95%CI		*P*	HR	95%CI		*P*	HR	95%CI		*P*
Age	Continuous	1.040	1.009	1.073	0.012	1.013	0.977	1.050	0.492	1.035	1.000	1.071	0.051
Sex	M vs. F (Ref)	3.157	0.932	10.690	0.065	1.481	0.403	5.448	0.554	1.309	0.350	4.901	0.689
Facial nerve palsy at diagnosis	Yes vs. No (Ref)	1.541	0.645	3.679	0.330								
Tumor size^a^	Continuous	1.250	1.065	1.467	0.006	1.215	1.029	1.435	0.022	1.185	1.005	1.398	0.044
Node metastasis^b^	Yes vs. No (Ref)	4.586	1.783	11.800	0.002					3.998	1.428	11.193	0.008
Tumor grade	High grade vs Others (Ref)	3.353	1.382	8.135	0.007	5.385	1.657	17.499	0.005				
Perineural invasion	Yes vs. No (Ref)	1.373	0.533	3.534	0.511								
Surgery	Yes vs. No (Ref)	0.408	0.171	0.975	0.044	0.255	0.080	0.817	0.021	0.932	0.332	2.613	0.893
Distant metastasis	Yes vs. No (Ref)	2.098	0.704	6.256	0.184								

^a^Tumor size: Determined by preoperative radiology

^b^Node metastasis: Clinical nodal status

We used clinical variables (tumor size and cN) in this analysis, because 11 patients did not undergo surgery

^c^Due to a possibility of potential correlation between lymph node metastasis and tumor grade (high grade) (*P* = 0.05), we separately constructed two multivariable models

*HR* Hazard ratio, *95%CI* 95% confidence interval

Considering the possible inter-correlations between variables, we performed multivariable analysis of significant variables (*P* < 0.1) from univariable analyses. In this analysis, we detected potential correlation between presence of lymph node metastasis and tumor grade (high grade) (*P* = 0.05); thus we separately constructed two multivariable models. Of note, initial surgical treatment was not associated with presence of distant metastasis, because of adenoid cystic carcinoma cases. As a result, we found that tumor size, nodal metastasis and tumor grade were independent prognostic factors for OS in bulky PGC. Interestingly, distant metastasis itself was not a significant predictor for overall survival in our series. This was due to the slow progression of metastatic diseases in adenoid cystic carcinomas and other non-high-grade tumors (long-term survivals with metastasis, *N* = 4 of 8 cases of distant metastasis).

## Discussion

One of the main findings in this study was that the risk of high-grade malignancy was increasing in proportion to primary tumor burden in PGC (Table 1). In bulky PGC, tumor size was one of the independent prognostic factors for survival, supporting the importance of early detection and diagnosis of PGC for improving patient survival and treatment outcome (Table 4). In addition, our data also demonstrated that lymph node metastasis at diagnosis and high tumor grade associated with poor survival outcomes, and functional preservation of the facial nerve was achieved in less than half of the cases (Tables 3 and 4). Overall, our results emphasize diagnosis and management of PGC at an early stage for better outcomes.

Many previous studies have reported that T status or primary tumor burden is a powerful prognostic indicator in salivary gland cancers [3, 4, 6, 7, 12]. Based on similar findings in our study, we tried to explain why advanced T status or large tumor is associated with poor prognosis in PGC. Indirectly, we found that advanced T status or large tumors in PGC had high risk of advancing to high-grade cancer and nodal metastasis, which can result in poor prognosis (less than 50% survival at 5 years) (Table 4, Fig. 2).

In addition, preservation of the facial nerve during surgery for PGC was relatively difficult because of the big size of primary tumors. In our experience of parotidectomy for malignant parotid tumors (in another study), 21.7% (31/143) of patients with malignancy parotid tumor showed temporary facial weakness and 14.4% (19/143) experienced permanent facial weakness [14], which was similar to previous reports [15–22]. However, anatomical preservation of the facial nerve was possible in only one-third of bulky PGC patients (Table 3) and functional facial weakness was observed in more than half of patients during clinical follow up (63.3%, 31 of 49 cases). Thus, patients with bulky PGC suffered from poor quality of life (facial weakness) due to tumor or surgery, along with poor prognosis.

Regarding the epidemiologic trend focusing on incidence of salivary gland tumors, Del Signore et al. reported that the rate of major salivary gland cancer measuring 0 to 2.0 cm in diameter has increased with time in the United States [5]. Meanwhile, the incidence of tumors larger than 2.0 cm was not increased, suggesting that early diagnosis and management of parotid tumors may be one reason for this shift (more tumors smaller than 2.0 cm are detected in the major salivary glands). Even with this trend, there is a need for some tools of early detection and early referral of parotid tumor patients to improve treatment and functional outcomes of PGC. It may facilitate this epidemiologic shift for the early diagnosis and public awareness of PGC, globally beyond some developed countries.

To find a solution, we have investigated screening programs in a variety of cancers. Self-examination has long been advocated for many malignancies. For example, since the first evidence of effectiveness of breast self-examination (BSE) in 1978 [23, 24], BSE has become a routine procedure for detecting breast cancer at an early stage. In other cancers, screening tools such as gastroscopy for stomach cancer [25], colonoscopy for colorectal cancer [26], and ultrasonography for thyroid cancer [27], have also had essential value for patients at risk of these cancers.

**Fig. 3:**
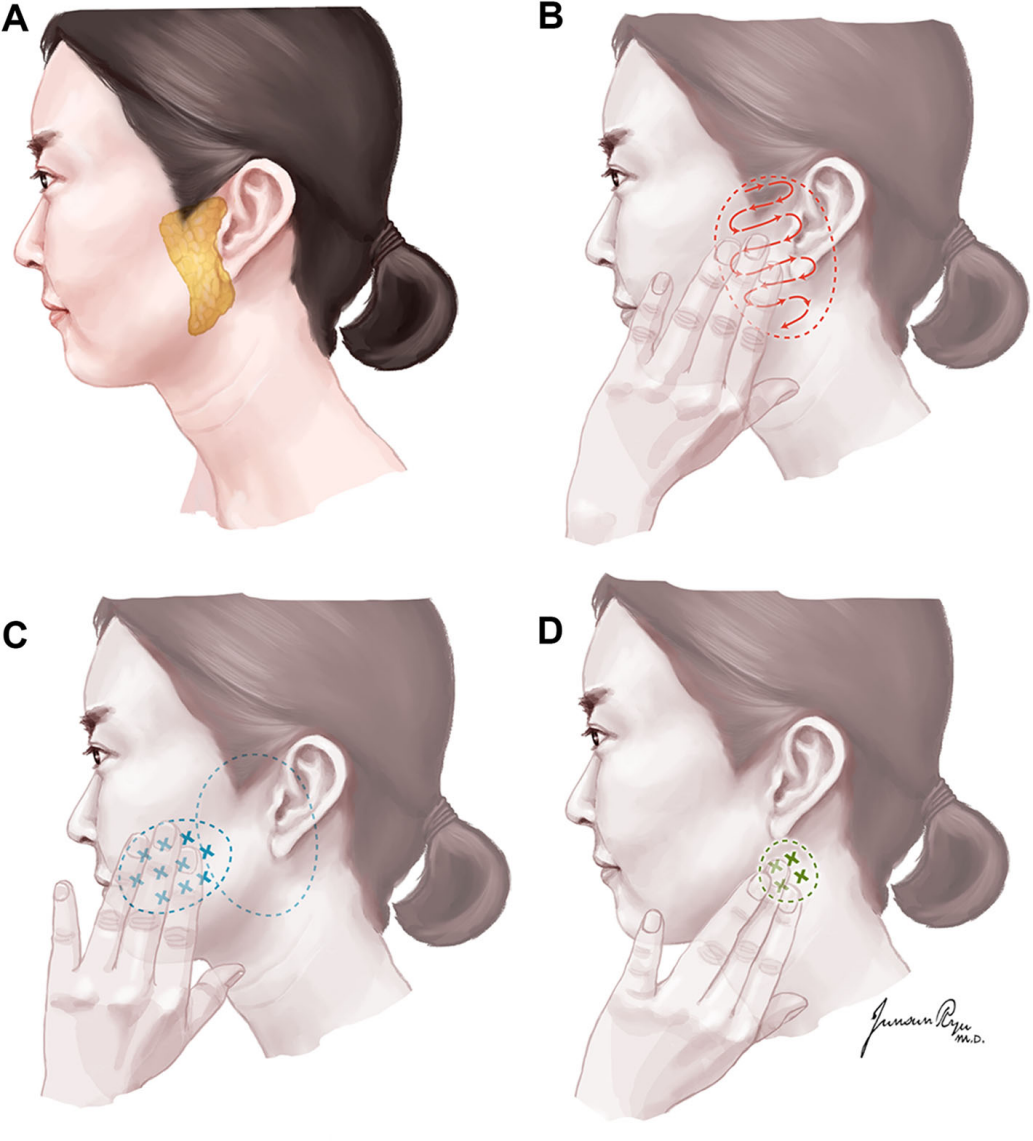
Proposed steps for self-examination of the parotid gland. **a** Understand the location of the parotid gland. **b** Palpate the parotid gland carefully to detect any lump. **c** Palpate the area from the parotid gland to mouth angle and nostril (accessory parotid gland). **d** Palpate the area between the jaw bone and the mastoid bone. Instruction: Normally, you can detect only bony structures [cheek bone (zygoma), jaw bone (mandible), and ear bone (mastoid bone)] around the parotid gland. If you feel any mass in the parotid self-examination, persistent for more than 2 weeks, you should visit a specialist for diagnosis

Fortunately, most of parotid tumors are located superficially around the ear, which can be easily palpated similar to the breast. In this context, we design a self-examination protocol for parotid tumor detection, using concepts of BSE. This is an easy, feasible and step-by-step protocol for self-examination of the parotid gland and self-detection of a parotid mass (Fig. 3). Although utility of the parotid self-examination in improving the detection rate of parotid tumor is not yet proven, it may play a role in public awareness of PGC, increasing early referral of parotid tumor patients for timely diagnosis and management.

Despite the potential beneficial effect of our self-examination protocol, its efficacy should be validated in future study. In addition, as the study collected clinical data retrospectively, each prognostic factor should be interpreted cautiously. Nevertheless, with these limitations, we believe that our study is meaningful as the first investigating the outcomes of a unique subset of bulky PGC and proposing a parotid self-examination for early diagnosis of PGC.

## Conclusion

In bulky PGC, lymph node metastasis at diagnosis and high tumor grade indicated poor survival outcomes, and functional outcomes of the facial nerve were suboptimal. Thus, a public effort seems to be necessary to decrease these patients with bulky PGC, and to increase patients’ self-awareness of their disease. As a way of early detection, we proposed a parotid self-examination tool to detect parotid gland tumors at an early stage, which is similar to breast self-examination. Our self-examination tool could serve as one reference to improve not only public awareness but also timely diagnosis and management of parotid tumors. However, it should be further validated through a prospective large study, in terms of specific methods, its clinical efficacy and impact on public awareness of disease.

## Supplementary Information

**Additional file 1: Supplementary File** Raw data for this study

## Data Availability

All data generated or analyzed during this study are included in this published article and its supplementary information file. Any administrative permissions are not required to access the raw data.

## Abbreviations

PGC: Parotid gland cancer
DFS: Disease-specific survival
OS: Overall survival
TNM staging: Tumor-node-metastasis staging
CT: Computed tomography
MRI: Magnetic resonance imaging
